# Tracking a decade of phase I clinical trials in Italy: trends and insights from national and european registries amid regulatory change

**DOI:** 10.3389/fphar.2025.1661186

**Published:** 2025-08-29

**Authors:** Eleonora De Paola, Luna Del Bono, Diego Alejandro Dri, Raffaella Maione, Sandra Petraglia, Fabrizio Galliccia, Giovanni Gori

**Affiliations:** ^1^ Clinical Trials Office, Italian Medicines Agency (AIFA), Rome, Italy; ^2^ Department of Pharmacy, School of Specialization in Hospital Pharmacy, University of Pisa, Pisa, Italy; ^3^ Clinical Pharmacology Centre for Drug Investigation, Pisa University Hospital, Pisa, Italy; ^4^ Pre Authorisation Department, Italian Medicines Agency (AIFA), Rome, Italy

**Keywords:** phase I, clinical trials, regulation (EU) 536/2014, Italy, CTIS, EudraCT

## Abstract

**Introduction:**

The global clinical research landscape has expanded significantly over the past decade, yet Europe has witnessed a decline in its share of clinical trial activity, especially in early-phase trials. This study aims to characterize the evolution of Phase I clinical trials in Italy from January 2015 through the completion of the European regulatory transition under Regulation (EU) 536/2014 (30 January 2025), by analyzing trends in trial submissions, sponsor typologies, study designs, and participant populations.

**Methods:**

A systematic extraction of data was conducted from the Italian *Osservatorio sulla Sperimentazione Clinica* (OsSC), European Union Drug Regulating Authorities Clinical Trials Database (EudraCT) and the EU Clinical Trials Information System (CTIS), covering all Phase I trials submitted to the Agenzia Italiana del Farmaco (AIFA; Italian Medicines Agency), the Competent Authority for clinical trials in Italy, between 1 January 2015, and 30 January 2025. Trials were categorized by sponsor type, authorization outcome, therapeutic area, study design, investigational medicinal product (IMP) classification, and participant population characteristics. Descriptive and comparative analyses were conducted.

**Results:**

A total of 1,051 Phase I clinical trials were submitted during the study period, accounting for 14.4% of all clinical trial applications in Italy. Commercial sponsors dominated (90.6%), and oncology was the leading therapeutic area (69.6%). The overall regulatory authorization rate was high, with no statistically significant difference between commercial and non-commercial sponsors (89.5% vs. 86.9%, respectively; p > 0.05). Trials exclusively involving healthy volunteers were limited (3.4%). Among the 3,777 IMPs analyzed, chemical compounds prevailed (66.1%), followed by biologics (28.7%) and Advanced Therapy Medicinal Products (ATMPs, 3.3%), the latter showing a steady increase over the decade. Non-commercial sponsor trials more frequently included pediatric and vulnerable populations, thus underlining their distinct contribution to underrepresented areas of clinical research.

**Conclusion:**

While Phase I trial activity in Italy remained stable over the past decade, structural imbalances persist—including limited non-commercial engagement, strong therapeutic concentration in oncology, and underuse of healthy volunteer models. Strengthening infrastructure for early development, supporting academic-led studies, and promoting broader inclusion in study populations may help consolidate recent progress and foster a more balanced national research ecosystem.

## 1 Introduction

Over the past decade, the global clinical research landscape has undergone remarkable growth and geographic shifts. Between 2013 and 2023, the total number of clinical trials worldwide increased by 38%, primarily driven by heightened activity in Asia and North America (IQVIA-EFPIA-VE, 2024). China alone doubled its commercial trial output and now accounts for 18% of global activity. In contrast, Europe’s share of global trials declined of 10% from 22% to 12%, largely due to regulatory fragmentation and extended trial start-up times (IQVIA-EFPIA-VE, 2024).

Phase I trials have been a central driver of global commercial clinical research growth, recording a 4.5% increase from 2013 to 2023, slightly outpacing the overall 4% growth of all clinical trials. However, within Europe, Phase I activity has contracted. In 2022 and 2023, Phase I trials represented 16% and 14% of European Economic Area commercial trials, compared to 39% and 41% globally (IQVIA-EFPIA-VE, 2024). A similar decline was observed in Phase II/III oncology trials, particularly affecting studies on cell and gene therapies, biosimilars, and rare diseases.

New global data confirms the persistence of these trends. The 2025 IQVIA Institute Report (IQVIA Institute for Human Data Science, 2025) shows a total number of industry-sponsored clinical trials stabilized at 5,318 — effectively returning to pre-pandemic levels (5,316 in 2019), despite a 17% decline from the 2021 peak. However, this normalization masks significant changes: emerging biopharmaceutical companies (EBPs) accounted for 63% of all trial starts and 65% of Phase I trials, surpassing large pharmaceutical sponsors across in all phases. Notably, pre-commercial EBPs initiated more than twice as many Phase I trials as large companies, reflecting their increasing involvement in early-stage innovation. At the same time, geographic polarization has continued: U.S.-headquartered sponsors led with 35% of global trial starts, followed closely by China at 30%, while Europe declined to 21%. Phase I trials have become a privileged testing ground for novel modalities, including multispecific antibodies, radioconjugates, and cell and gene therapies, particularly in oncology. These accounted for 32% of oncology Phase I starts in 2024, marking a threefold increase over the past decade and being predominantly driven by EBPs (IQVIA Institute for Human Data Science, 2025).

The European regulatory landscape has simultaneously evolved. The adoption of Regulation (EU) No. 536/2014 (European Union, 2014) marked a pivotal change, with the aim of harmonizing the assessment and supervision of clinical trials across Member States. The Regulation came into full effect in 2022 and introduced the Clinical Trials Information System (CTIS) to streamline submissions, evaluations and reporting, replacing the previous Directive 2001/20/EC (Council of the European Union, 2001) framework.

This transition, which was completed in January 2025, is intended to facilitate multinational trials, strengthen participant safety (Gefenas et al., 2017), and increase transparency—though the latter remains limited for Phase I studies (De Mey, 2022).

Despite these reforms, the decline in Phase I activity within Europe poses a strategic threat. Phase I trials are foundational to drug development, offering critical insights into safety, pharmacokinetics, pharmacodynamics, and dosing strategies (Iasonos and O’Quigley, 2021; Thall et al., 2024). They also catalyze innovation by enabling the early exploration of novel modalities such as biologics, gene therapies, and immunotherapies, while fostering the growth of specialized infrastructures and expert research teams (Kurzrock and Hong, 2024).

Italy provides an illustrative case within this evolving context. According to the 21st National Report on Clinical Trials of Medicines in Italy (AIFA, 2025a), released by the Agenzia Italiana del Farmaco (AIFA; Italian Medicines Agency), the Competent Authority for clinical trials in Italy, the country saw 818 authorized clinical trials in 2021, peaking before declining to 611 in 2023. While these figures encompass all phases and therapeutic areas, Phase I trials merit particular attention due to their complexity and strategic importance. Regulatory initiatives such as the AIFA Determina 809/2015 (AIFA, 2015) established stringent quality standards for Phase I units, with the aim of improving the conduct of early-phase trials. However, unlike expectations, these measures did not result in substantial growth in Phase I studies in the subsequent years. Several factors may explain this limited impact: the high operational costs associated with compliance to Determina 809/2015 may have deterred some centers from pursuing certification; the absence of complementary national incentives (e.g., funding mechanisms, streamlined start-up processes) may have hindered broader adoption; and the persistence of structural barriers—including the limited availability of healthy volunteer registries and fragmented early-phase trial infrastructure—likely continued to constrain growth. Therefore, despite the regulatory effort to promote a stronger Phase I ecosystem, the quantitative expansion of trials remained modest.

In light of these dynamics, it is interesting to critically appraise the Italian Phase I landscape.

This study is based on data extracted from clinical trial applications submitted to AIFA, which maintains the OsSC (Osservatorio Nazionale sulla Sperimentazione Clinica) database and has full access to trials submitted via EudraCT (European Union Drug Regulating Authorities Clinical Trials Database) and CTIS (Clinical Trials Information System). The analysis covers the period from January 2015 to January 2025 and aims to assess trends in Phase I clinical trials conducted in Italy. It examines the classification features, therapeutic focus, sponsor type, and population characteristics of these trials. By contextualizing national patterns within a broader international framework, we aim to highlight areas of strength, identify gaps in competitiveness, and uncover opportunities for regulatory and institutional alignment.

## 2 Materials and methods

### 2.1 Data sources

**FIGURE 1:**
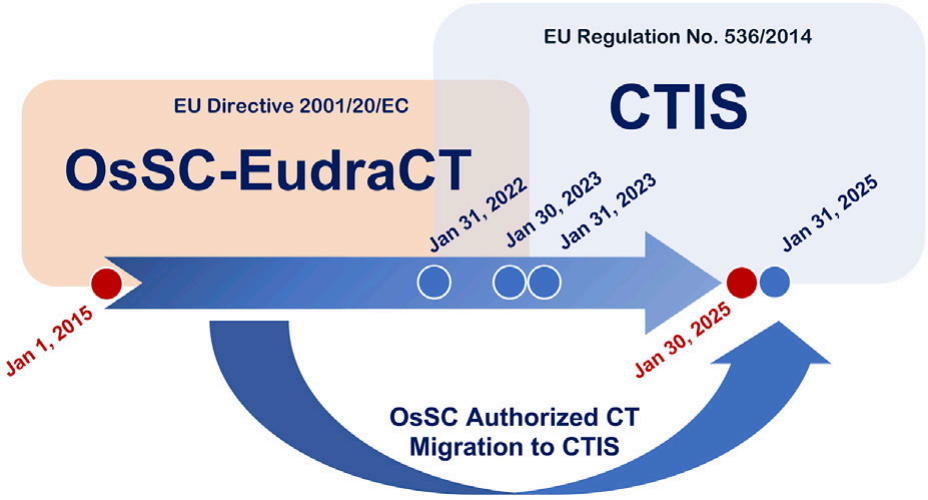
Time pattern of OsSC/EudraCT and CTIS platform usage for clinical trial application submissions to the Italian Competent Agency (AIFA). The *red circles* indicate the start (1 January 2015) and end (30 January 2025) of the data collection period for the present analysis. The *Blue circles* mark key milestones in the implementation of EU-CTR No. 536/2014: – 31 January 2022: the EU-CTR comes into force and the “transition period” begins; – 30 January 2023: the deadline for submitting new clinical trial applications via the AIFA Clinical Trials Observatory (OsSC) according to the EU Directive 2001/83/EC; – 31 January 2023 the Clinical Trials Information System (CTIS) becomes mandatory for all new applications; – 31 January 2025: the deadline for migrating all trials previously authorized via OsSC to CTIS, marking the official end of the EU-CTR “transition period”.

This study systematically analyzed all Phase I clinical trial applications submitted to AIFA between 1 January 2015, and 30 January 2025, under different regulatory legislation (Figure 1). The data supporting the findings of this study were extracted from regulatory submission platforms managed by AIFA and the European Medicines Agency (EMA), based on the period and applicable legislation considered, and reflect the characteristics entered by the sponsors in the platforms at the time of submission. Aggregated data used in this study were obtained through structured queries under the supervision of AIFA and are not publicly available in their entirety due to regulatory confidentiality constraints. In particular, data were retrieved from three regulatory platforms:

• OsSC (https://www.aifa.gov.it/sperimentazioni-cliniche) and EudraCT (https://www.clinicaltrialsregister.eu/ctr-search/search) for trials submitted under Directive 2001/20/EC (Council of the European Union, 2001) from 2015 to 30 January 2023.
• CTIS (https://euclinicaltrials.eu/search-for-clinical-trials/?lang=en) for trials submitted under Regulation (EU) No. 536/2014 from 31 January 2022, onward. From 31 January 2023, CTIS became the sole platform for all new submissions, completing the regulatory transition.

### 2.2 Inclusion and exclusion criteria

All Phase I studies were included, encompassing:

• Standalone Human Pharmacology Phase I: First-in-Human (FIH), Bioequivalence, Other Human Pharmacology (e.g., new indications/associations/formulations for marketed drugs)
• Integrated Phase: I/II, I/III, I/II/III, and I/III/Bioequivalence studies as per Italian Ministerial Decree of 27 April 2015 (Italian Ministry of Health, 2015).

All other clinical trials, Phase 2, 3 and 4 were excluded from the analysis.

Phase 1 trials were included regardless of regulatory outcome (authorized, authorized with conditions, not authorized, withdrawn, under evaluation, lapsed and not valid). If a trial was resubmitted after withdrawal or rejection, only the latest application was considered. This approach was adopted to capture the most up-to-date regulatory status of each study and to accurately determine which trials were effectively authorized and conducted within the Italian territory. Trials that transitioned from the EU-CTD to the EU-CTR via CTIS (EMA, 2024) were excluded as they are clinical trials authorized under the Directive 2001/20/EC and were already included in the data extracted from the OsSC and EudraCT. Trials in which Italy was added as a Member State under Article 14 of the CTR (European Union, 2014) were also excluded in line with the methodology used by EMA in its periodic reports on clinical trials. Submissions of Standalone IMPD-Q (Investigational Medicinal Product Dossier–Quality) (EMA, 2023; EMA, 2025a) were excluded from the trial count but included in the IMP analysis.

### 2.3 Data extraction and validation

Structured database queries were executed using an Oracle-based database management system. The Extracted datasets were then exported into Excel (Version16.54; ^©^2021 Microsoft) spreadsheets for analysis. Data validation was performed through double-checking by two independent experts under AIFA Clinical Trials Office supervision. Additionally, a simple random sampling of 20% of the dataset was conducted to ensure consistency and reproducibility with the official registry data.

### 2.4 Variables collected

The following data were extracted for each trial:

• Submission platform (OsSC, EudraCT or CTIS);
• Trial phase classification (standalone Phase 1, FIH or bioequivalence, and integrated Phase I/II, I/III, I/II/III);
• Sponsor type (commercial or non-commercial);
• Participant type (healthy volunteers, patients or both, with vulnerable populations considered within these groups);
• Age and sex inclusion criteria;
• Therapeutic area (according to the Medical Subject Headings, MeSH-based classification)^1^;
• Investigational Medicinal Product (IMP) category^2^: chemical, biological, Advanced Therapy Medicinal Product (ATMP), or combinations of chemical/biological.

Predefined filters were used to extract eligible trials (further details of Query Strategy could be requested as Supplementary Materials).

### 2.5 Data analysis strategy

Descriptive statistics were used to summarize the characteristics of Phase I trials. Categorical variables were mostly presented as n/N (%), when appropriate.

Differences between categorical variables (e.g., sponsor type vs. participant type) were assessed using chi-square tests of independence. All analyses were performed using SPSS software (Version 29, IBM Corp., Armonk, NY, United States). A quadratic regression analysis was performed to evaluate a concave-down (inverted U-shaped) trend in the number of clinical trials across three time periods: pre-pandemic, pandemic, post-pandemic/regulation implementation, with a peak expected during the pandemic period.

A p-value less than 0.05 was considered statistically significant.

### 2.6 Data Governance and Ethics

This study is based on the analysis of clinical trial application data submitted to regulatory platforms. No clinical data or individual patient-level information were accessed or analyzed. The study did not involve any intervention, interaction with study participants, or the use of personal data. Therefore, ethical approval by a Research Ethics Committee was not required.

## 3 Results

### 3.1 Clinical trial submissions overview

**FIGURE 2:**
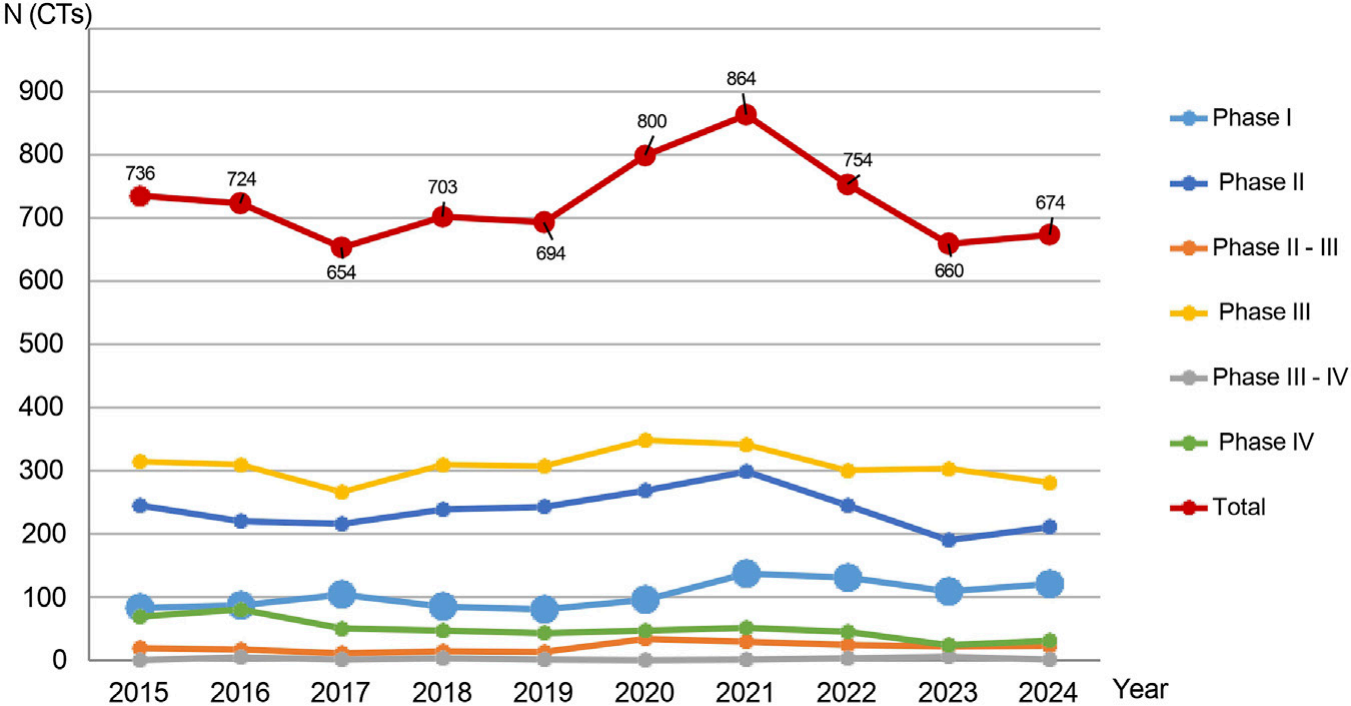
Clinical studies submitted to the AIFA per year and per phase (n = 7,263). Number of clinical trials (CTs) submitted to AIFA per year and phase (n = 7,263). Clinical trials submitted in January 2025 (n = 43) were not taken into account for the purposes of this graph, i.e., the performance of a phase over the course of a year.

**FIGURE 3:**
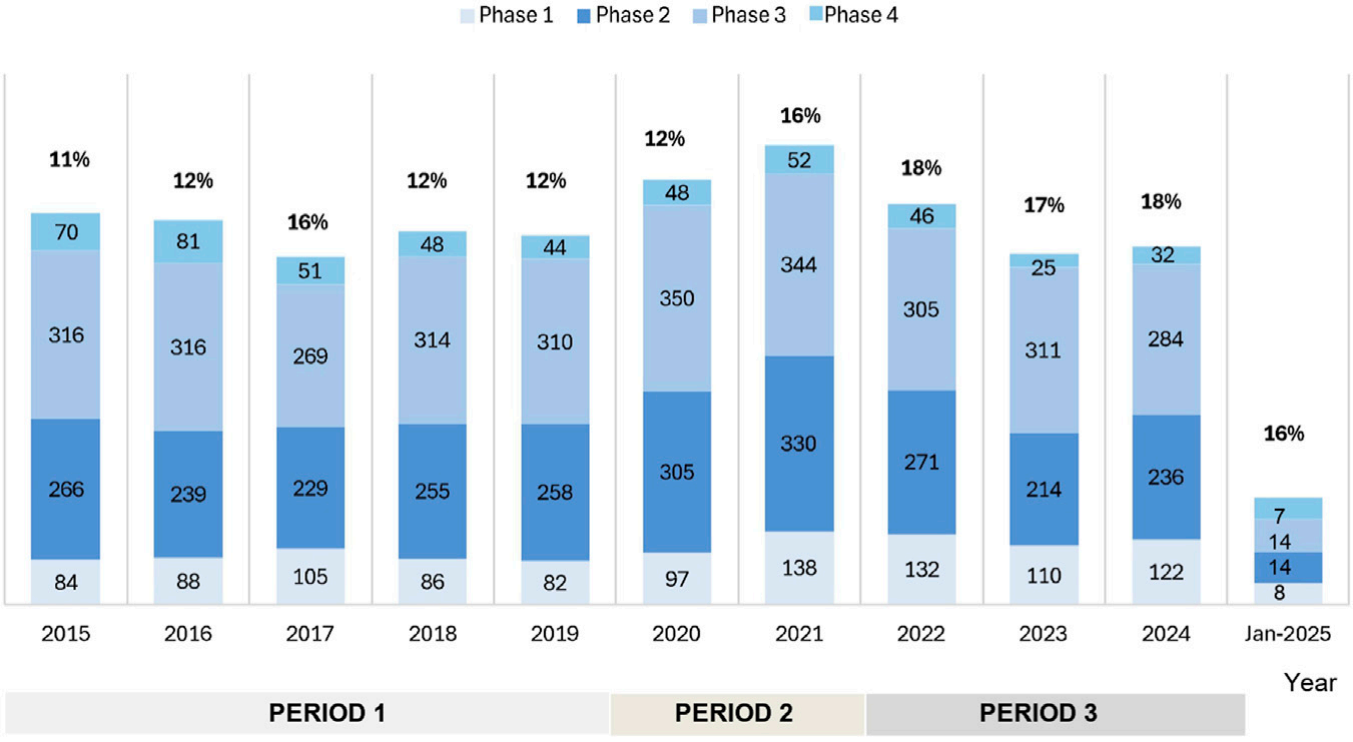
Annual number of Phase I clinical trials submitted in Italy from 2015 to January 2025. For each year, the percentage (n%) indicates the proportion of Phase I trials relative to the total number of clinical trials submitted in the same year. A temporal analysis revealed a non-linear trend in Phase I activity across three defined periods: Period 1 (2015–2019, pre-pandemic), Period 2 (2020–2021, COVID-19 pandemic), and Period 3 (2022–2024, post-pandemic and after the implementation of Regulation (EU) 536/2014).

Between 1 January 2015, and 30 January 2025, a total of n = 7,306 clinical trials were submitted to AIFA. Of these, n = 5,950 (81.4%) were submitted via the OsSC platform/EudraCT, and n = 1,356 (18.6%) via CTIS. The number of annual submissions ranged from n = 654 trials in 2017 to a peak of n = 864 trials in 2021, the final year before the CTIS was fully enforced (Figure 2). The most common type of study was Phase III (n = 3,102; 42.4%), followed by Phase II (n = 2,400; 32.8%), Phase I (n = 1,051; 14.4%), and Phase IV (n = 505; 7%). Integrated Phase II/III accounted for n = 217 (3%), while Phase III/IV trials accounted for n = 31 (0.4%). The proportion of Phase I trials, relative to the total volume of clinical trials, varied over time, with annual rates of change differing from year to year (Figure 3). When three distinct periods were defined—Period 1 (2015–2019, pre-pandemic), Period 2 (2020–2021, COVID-19 pandemic), and Period 3 (2022–2024, post-pandemic and following the implementation of Regulation (EU) 536/2014)—an initial increase in both the absolute number and proportion of Phase I trials was observed from Period 1 to Period 2, followed by a subsequent decline in Period 3. Quadratic regression modelling confirmed the statistical significance of this non-linear trend (p = 0.012), indicating that changes in early-phase activity evolved progressively over time rather than occurring through abrupt transitions.

### 3.2 Authorization outcome

**FIGURE 4:**
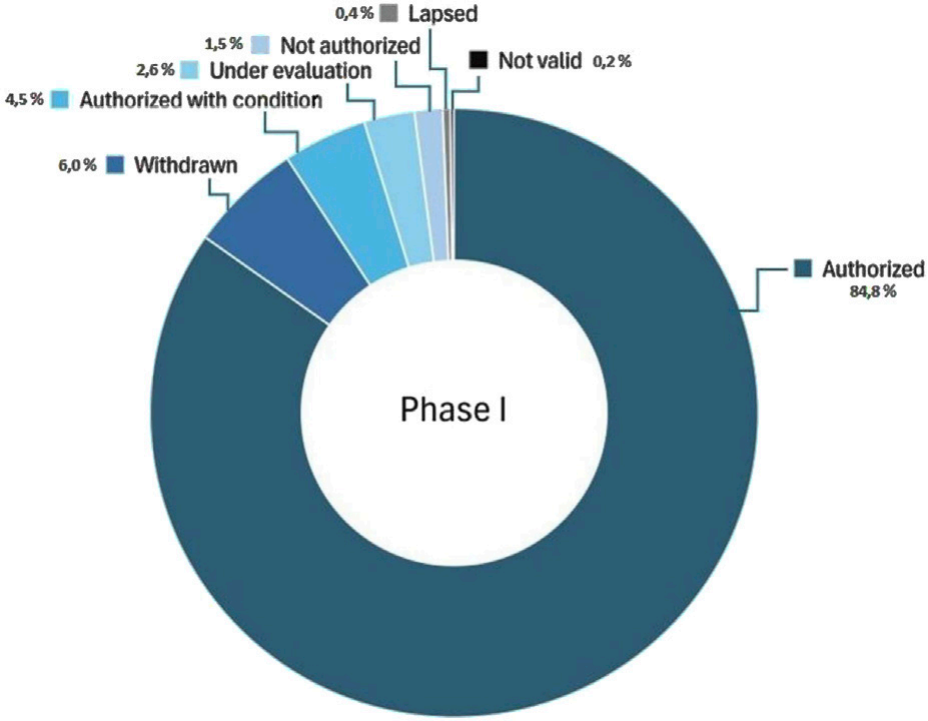
Percentage Distribution of Phase I Clinical Trial Outcomes in Italy (2015–2025). This shows the percentage of Phase I clinical trials submitted in Italy between January 2015 and January 2025, categorized by outcome: authorized, authorized with conditions, not authorized, withdrawn by sponsor, lapsed, not valid, and under evaluation.

Of the 1,051 Phase I trials submitted, n = 891 (84.8%) were authorised unconditionally and n = 473 (4.5%) conditionally. Figure 4 displays the entire dataset, regardless of sponsor type. A stratified analysis by sponsor typology reveals that commercial sponsors accounted for the majority of approvals, receiving n = 812 (77.3%) unconditional and n = 40 (3.8%) conditional authorizations. Non-commercial sponsors, who represented only 9.4% of submissions (n = 99/1,051), received n = 79 (7.5%) unconditional and n = 7 (6.6%) conditional approvals. In percentage terms, 89.5% of trials from commercial sponsors and 86.9% from non-commercial sponsors were approved, with no statistically difference between the two groups (p > 0.05). Negative or unresolved outcomes were infrequent but notable: n = 16 trials (1.5%) were not authorized, including n = 12 submitted by commercial sponsors and n = 4 by non-commercial ones. All n = 28 (2.6%) trials that were still under evaluation at the time of the analysis were submitted by commercial sponsors.

### 3.3 Design classification

**FIGURE 5:**
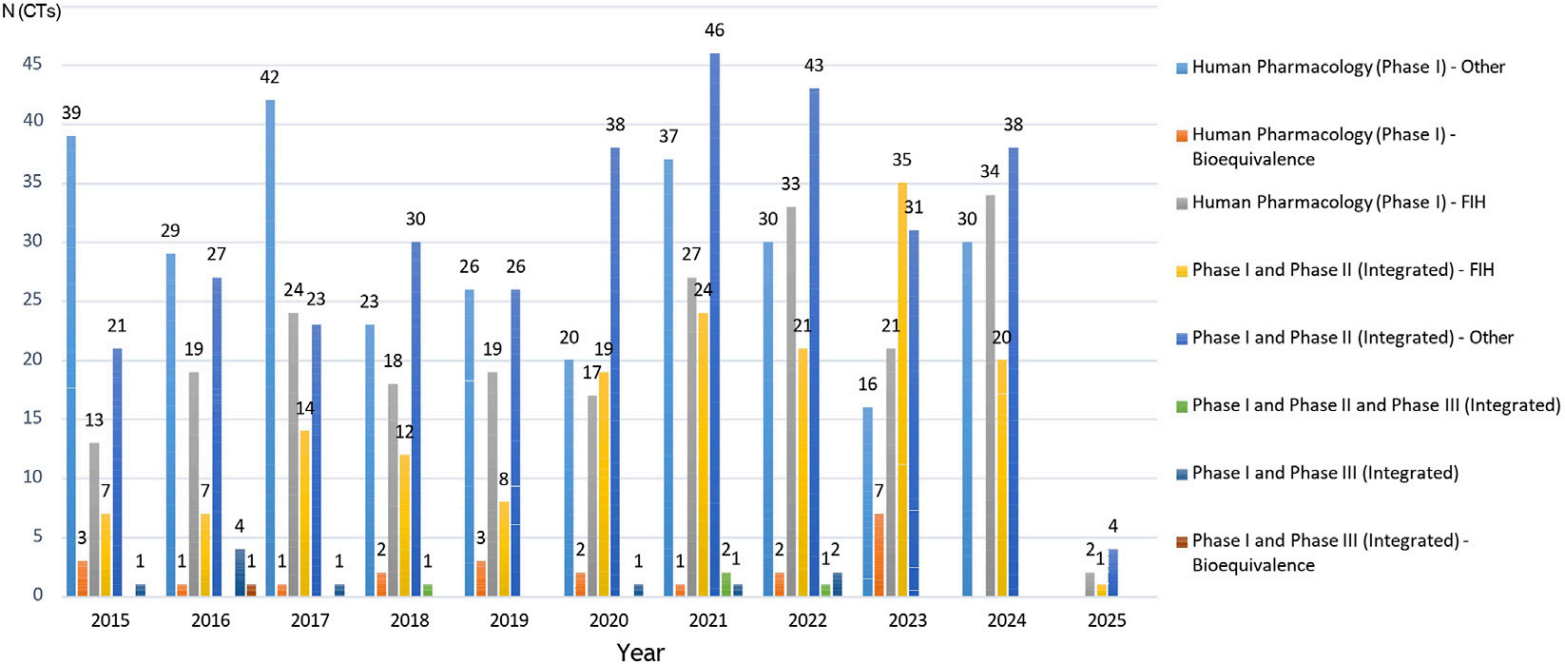
Annual distribution of Phase I clinical trial classifications in Italy (January 2015–January 2025). This graph shows how these trends have changed over time for different types of Phase I study, including standalone or integrated Phase I (with bioequivalence, Phase II and/or Phase III).

Among the 1,051 Phase I trials, integrated Phase I/II studies classified as “Other” were the most common (n = 327; 31.1%), followed by standalone Phase I–Other (n = 292; 27.8%), First-in-Human (FIH) trials (n = 227; 21.6%), and Bioequivalence studies (n = 23; 2.2%). Integrated FIH trials accounted for 16.0% (n = 168) and Phase I/III and I/II/III designs remained infrequent (n = 14; 1.3%) (Figure 5).

### 3.4 Sponsor typology

Commercial sponsors dominated the landscape, promoting 90.6% (n = 952/1,051) of all Phase I trials. Non-commercial entities, including universities and hospitals, accounted for just 9.4% (n = 99/1,051) (Table 1). The distribution of Phase I trial classifications was similar for both commercial and non-commercial sponsors (Table 2). In both groups, the most common category was Integrated Phase I/II–Other, accounting for 31.0% (n = 295) of commercial trials and 32.3% (n = 32) of non-commercial trials. This was followed by Phase I–Other studies, representing 27.8% (n = 265) and 27.3% (n = 27) of trials sponsored by commercial and non-commercial entities, respectively. Standalone FIH trials also featured prominently, accounting for 21.4% (n = 204) of trials sponsored by commercial entities and 23.2% (n = 23) of those sponsored by non-commercial entities. Integrated FIH designs were slightly less frequent, but still notable at 16.2% (n = 154) and 14.1% (n = 14) of trials sponsored by commercial and non-commercial entities, respectively.

**TABLE 1:** Annual distribution of Phase 1 clinical trials (CTs) by sponsor type. The table reports the yearly number (n) of Phase 1 CTs submitted to AIFA, categorized by sponsor type (commercial or non-commercial), along with the corresponding proportions (%) relative to the total number (N) of CTs submitted each year.

Year	Total CTs	Commercial		Non-commercial	
	N	n	%	n	%
2015	84	76	90.5	8	9.5
2016	88	83	94.3	5	5.7
2017	105	84	80	21	20
2018	86	76	88.4	10	11.6
2019	82	79	96.3	3	3.7
2020	97	87	89.7	10	10.3
2021	138	127	92	11	8
2022	132	118	89.4	14	10.6
2023	110	101	91.8	9	8.2
2024	122	114	93.4	8	6.6
2025 (Jan)	7	7	100	0	0
Total	1,051	952	90.6	99	9.4

**TABLE 2:** Classification of Phase I clinical trials by sponsor type. The table reports the number (n) and percentage (%) of Phase I trial designs, categorized by sponsor type (commercial or non-commercial). Percentages refer to the total number of trials (N) within each trial design category. *FIH: First-in-Human.*

Phase I classification	Total CTs	Commercial		Non-commercial	
	N	n	%	n	%
Phase I and Phase II (Integrated) - Other	327	295	31	32	32.3
Human Pharmacology - Other	292	265	27.8	27	27.3
Human Pharmacology - FIH	227	204	21.4	23	23.2
Phase I and Phase II (Integrated) - FIH	168	154	16.2	14	14.1
Human Pharmacology - Bioequivalence	22	20	2.1	2	2
Phase I and Phase III (Integrated)	11	10	1.1	1	1
Phase I and Phase II and Phase III (Integrated)	3	3	0.3	0	0
Phase I and Phase III (Integrated) - Bioequivalence	1	1	0.1	0	0
Total	1,051	952	100	99	100

### 3.5 Study population

Among the 1,051 registered Phase I trials, three-quarters involved adult (18–64yo) and elderly (65+ yo) participants. Only 8% of trials were restricted exclusively to the 18–64 age range. Pediatric populations (0–17yo) were exclusively included in a small part of trials, while mixed-age studies (0–17yo and 18–64yo with or without 65+ yo) represented 11%. (Table 3). A different trend in age distribution was observed when comparing sponsor types: commercial sponsors were more likely to include adult and elderly subjects (n = 810/952; 85% vs. n = 65/99; 65%, p < 0.001), whereas non-commercial sponsors more often enrolled pediatric participants (n = 34/99; 34.3% vs n = 142/952; 14.9%, p < 0.001).

**TABLE 3:** Demographic and participants features of population enrolled in Phase I clinical trials. The table presents the number (n) and percentage (%) of trials, stratified by age group, sex, and population type.

Demographics	Total CTs	
	n	%
Age, years		
18–64, 65+	791	75
18–64	84	8
0–17, 18–64, 65+	37	3.5
0–17, 18–64	71	7
0–17	68	6.5
Total	1,051	100
Sex		
Female, Male	992	94.4
Male	42	4
Female	17	1.6
Total	1,051	100
Participants		
Patients, Vulnerable Populations (VPs)	879	83.6
Patients	126	12
Healthy Volunteers (HVs)	46	4.4
HVs, VPs	36	3.4
HVs, Patients, VPs	10	1.0
Total	1,051	100

In terms of sex, the majority of studies enrolled both males and females. Male-only studies accounted for 4%, and female-only for 1.6% (Table 3). No significant difference in sex distribution was found between sponsor types (p > 0.05), although non-commercial sponsors had a slightly higher proportion of female-only trials.

Most studies (n = 1,005/1,051; 95.6%) enrolled patients, 83.6% of which included one or more vulnerable groups (Table 3). Only n = 46/1,051 trials (4.4%) involved healthy volunteers (HVs), either exclusively or in combination with patients. As for the different patterns of HV involvement, 36 out of 46 trials (3.4% of all Phase 1 trials) were conducted exclusively with healthy volunteers (classified as either non-vulnerable or vulnerable), and were almost entirely commercially sponsored (91.7%)

Most of the n = 36 HV-only studies were bioequivalence (n = 15; 41.7%), followed by other Human Pharmacology studies (n = 11; 30.6%) and FIH trials (n = 9; 25.0%). Of the FIH studies, three adopted an integrated Phase I/II design, while one was classified as an integrated Phase I/II–other. Thirteen out of 36 studies involved vulnerable participants, typically individuals unable to consent or elderly individuals.

Most HV studies (n = 30/46; /65.2%) involved adults (18–64yo), with the rare inclusion of pediatric (n = 3/46; 6.5%), elderly subjects (n = 12/46; 26.1%) or mixed category (n = 1/46; 2.2%). When stratified by sponsor type and excluding HV-only trials (n = 36), the analysis of the remaining n = 1,015 trials showed that vulnerable patients were enrolled in 91.2% (n = 808/886) of commercial trials and in 81.2% (n = 78/96) of non-commercial trials.

Among the n = 900 Phase I trials that reported the inclusion of specific vulnerable populations (either alone or in combination with other vulnerable categories), n = 796 trials (88.4%) involved women of childbearing potential (WCBP) who were using contraception, while n = 47 trials (5.2%) included WCBP who were not using contraception. A total of n = 139 trials (15.5%) enrolled participants who were incapable of providing consent. Trials conducted in emergency situations were rare, accounting for only n = 7 cases (0.7%). Some trials presented complex combinations of vulnerabilities. When analyzing differences by sponsor type, non-commercial sponsors were found to be more likely to include participants incapable of providing consent. Furthermore, trials enrolling both WCBP and subjects incapable of providing consent were significantly more common among non-commercial sponsors (n = 19/96, 19.8%) versus commercial sponsors (n = 77/919; 8.4%) (p < 0.001).

Overall, the most represented age group (18–64 and 65+ yo) showed a significant difference by sponsor (n = 739/952; 77.6% commercial versus n = 52/99; 52.5% trials non-commercial trials, p < 0.001). Commercially sponsored trials favored these age groups, which are likely to reflect a focus on adult diseases and market-driven priorities.

Non-commercial sponsors had relatively more pediatric trials, but these differences were not statistically significant. Similarly, sex-based enrollment differences (e.g., n = 3/99; 3.0% male-only non-commercial vs. n = 14/952; 14.7% commercial) were also non-significant (p > 0.05).

### 3.6 Therapeutic area

The majority of the n = 1,051 Phase I trials focused on just a few therapeutic areas. Given this concentration, the analysis focused on the ten most frequent categories, collectively accounting for 94% (n = 988/1,051) of all Phase I trials submitted during the study period (Table 4). This approach highlighted meaningful patterns while excluding underrepresented areas with limited analytical weight.

**TABLE 4:** Top ten therapeutic areas represented in Phase I clinical trials. The table presents the number (n) and percentage (%) of Phase I trials across the ten most represented therapeutic areas, stratified by sponsor type (commercial or non-commercial). Percentages refer to the total number of trials (N) within each therapeutic area. (*) Total percentages are calculated across the entire sponsor group.

	Therapeutic area	Total CTs	Commercial		Non-commercial	
		N	n	%	n	%
**1**	Neoplasms diseases	731	682	93.3	49	6.7
**2**	Hemic and Lymphatic diseases	76	62	81.6	14	18.4
**3**	Nervous System diseases	37	32	86.5	5	13.5
**4**	Immune System diseases	27	22	81.5	5	18.5
**5**	Nutritional and Metabolic diseases	23	18	78.3	5	21.7
**6**	Respiratory Tract diseases	22	18	81.8	4	18.2
**7**	Musculoskeletal diseases	22	19	86.4	3	13.6
**8**	Congenital, Hereditary, Neonatal diseases and Abnormalities	19	17	89.5	2	10.5
**9**	Cardiovascular diseases	16	14	87.5	2	12.5
**10**	Skin and Connective Tissue diseases	15	15	100	0	0
	Total ^(*)^	**988**	**899**	**91**	**89**	**9**

Analyzing the subset of trials conducted exclusively in HVs, the therapeutic areas represented included: Nutritional and Metabolic Diseases (n = 7/36), Nervous System Disorders (n = 6/36), Digestive System Diseases (n = 3/36), Infectious Diseases (n = 7/36), Cardiovascular and Respiratory Conditions (n = 4/36), and Oncology (n = 1/36; an atypical FIH study).

**FIGURE 6:**
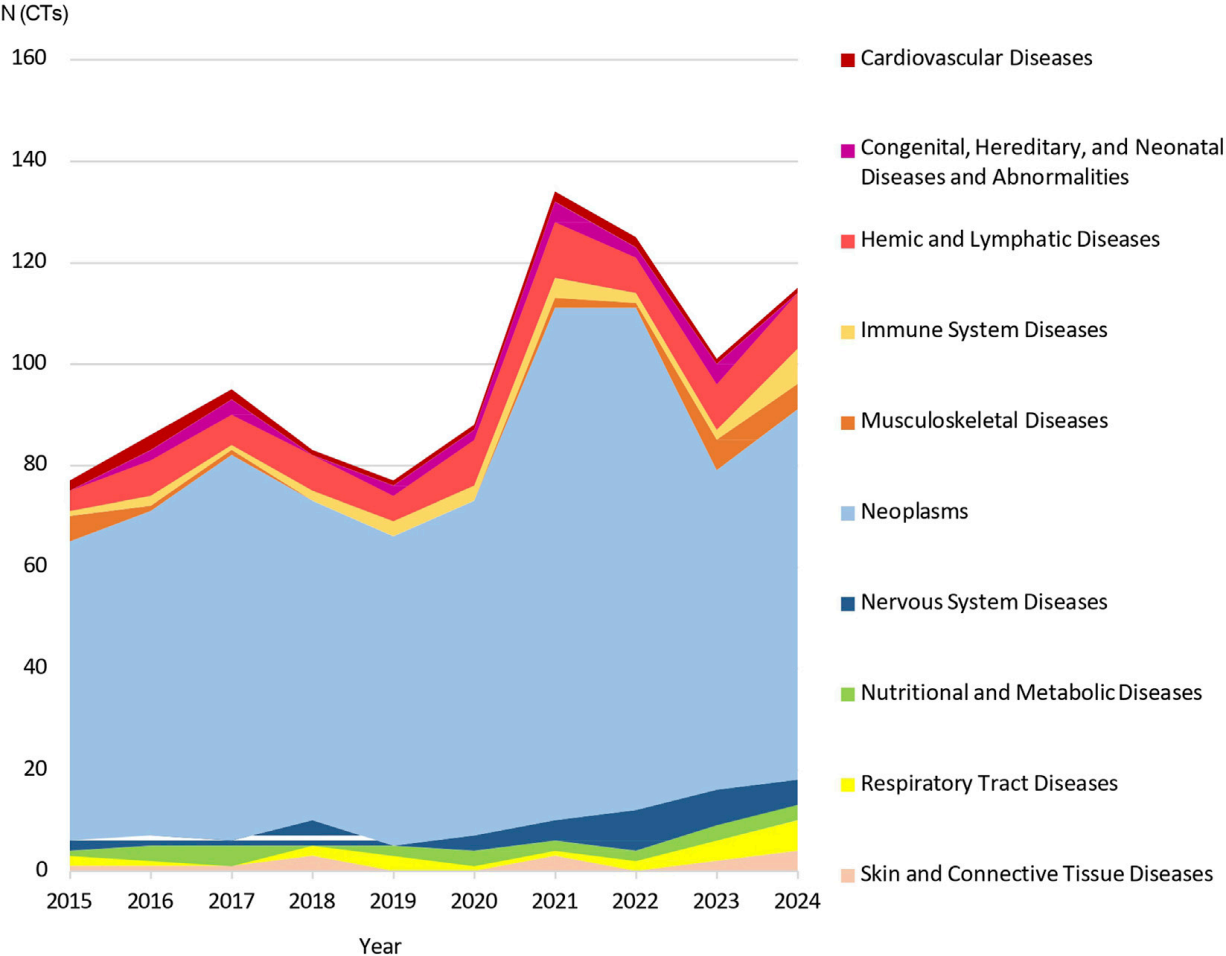
Stacked area chart illustrating the temporal distribution of Phase I clinical trials by therapeutic area (2015–2024). The chart displays the cumulative annual number (N) of trials across the top 10 therapeutic areas. The height of each colored band represents the relative contribution of that specific area to the total number of trials submitted each year. Phase 1 trials submitted in January 2025 were not considered when creating this graph.

As for the yearly trend, Oncology consistently dominated Phase I research, with peaks in 2021 (n = 101) and 2022 (n = 99), which together accounted for 27.4% of all oncology trials. Hemic and Lymphatic system trials ranged from 4 to 11 per year, with peaks in 2021 and 2024 (n = 11 each). Nervous system diseases showed steady activity throughout the decade, peaking in 2022 (n = 8) and representing over 20% of trials within this category. Immune system diseases displayed a gradual upward trend, reaching a peak of n = 7 trials in 2024. Musculoskeletal diseases increased in 2023 (n = 6) and 2024 (n = 5), with these 2 years alone accounting for 50% of all Phase I trials in this area over the ten-year period. In contrast, activity in the respiratory and cardiovascular areas fluctuated with no discernible trend (Figure 6).

#### 3.6.1 Stratification by age

The vast majority of studies included both adult and elderly participants, as observed in Oncology, where n = 604/731 trials (82.6%) participants aged the 18–64 and 65 + yo. Similar patterns were found in Hematologic diseases, with n = 48/76 trials (63.2%) including both age groups and in Nervous System diseases, with n = 11/37 trials (29.7%). In Immune System diseases, with n = 14/27 trials (51.9%) included both age groups. These figures highlight an emphasis on adult and elderly populations in early-phase research.

Paediatric involvement (0–17 yo) was limited overall, being more frequently observed in trials on Musculoskeletal diseases (n = 13/22; 59.1%), Congenital and Hereditary disorders (n = 8/19; 42.1%), and Oncology (n = 63/731; 8.6%). In other therapeutic areas, such as Hematologic diseases, Immune diseases and Nervous System diseases, paediatric participants were occasionally included, usually as part of broader age group combinations.

#### 3.6.2 Stratification by sex

The majority of trials (n = 992/1,051; 94.4%) enrolled participants of both sexes. Studies involving only females or males were rare. Of Oncology trials, 1.9% (n = 14/731) were male-only. Musculoskeletal diseases trials had the highest share of male-only studies (n = 5/22; 22.7%). Female-only studies appeared in some Oncology (n = 15/731; 2.0%) and Female Urogenital diseases (n = 1/1; 100%) trials.

#### 3.6.3 Stratification by vulnerable subjects

**FIGURE 7:**
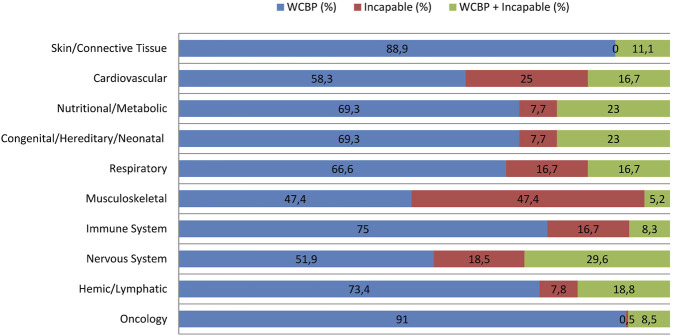
Distribution of Vulnerable Subgroups across top ten Therapeutic Areas. This figure illustrates the percentage distribution of three vulnerable population subgroups across the ten leading therapeutic areas.

Oncology trials showed the highest inclusion of vulnerable population (n = 656 trials), with WCBP participation in n = 596/656 trials (91%), with minimal inclusion of incapable individuals (n = 3/656; 0.5%) and few trials including both (n = 57/656; 8.5%). Musculoskeletal disease trials had the highest inclusion of participants incapable of consent (n = 9/19; 47.4%), followed by Cardiovascular (n = 3/12; 25%) and Nervous System trials (n = 5/27; 18.5%). Other therapeutic areas, such as Respiratory (n = 3/18; 16.7%) and Immune System diseases (n = 4/24; 16.7%), also showed moderate inclusion of cognitively impaired individuals. Trials involving both vulnerable subgroups were most frequent in Nervous System (n = 8/27; 29.6%) (Figure 7).

#### 3.6.4 Stratification by sponsor typology (commercial vs non-commercial)

Commercial sponsors dominated across all therapeutic areas, being responsible for n = 952 Phase I trials (90.6%), while non-commercial sponsors submitted just n = 99 trials (9.4%), and their level of engagement varied significantly by therapeutic area. In Oncology, the most represented field, non-commercial sponsorship accounted for just 6.7% of trials (n = 49/731). Similarly, Digestive diseases and Cardiovascular diseases showed low levels of academic involvement, with 9.1% (n = 1/11) and 12.5% (n = 2/16) non-commercial sponsorship, respectively.

In contrast, some therapeutic areas exhibited higher-than-average academic participation. Nutritional and metabolic diseases had the highest proportion of non-commercially sponsored trials, at 21.7% (n = 5/23). This was followed by immune system diseases at 18.5% (n = 5/27), closely mirrored by Hemic and Lymphatic diseases at 18.4% (n = 14/76), and Nervous System diseases at 13.5% (n = 5/37).

No non-commercially sponsored Phase I trials were recorded in the following nine therapeutic categories: Skin and Connective Tissue diseases, Bacterial Infections and Mycoses diseases, Analytical-Diagnostic-Therapeutic Techniques and Equipment, Immune System Processes, Reproductive and Urinary Physiological Phenomena, Metabolism diseases, Digestive System and Oral Physiological Phenomena, Otorhinolaryngologic diseases, Hormonal diseases.

### 3.7 Investigational medicinal product

#### 3.7.1 Yearly distribution by category

Between 2015 and 2025, n = 3,777 IMP entries were recorded in Phase I trials in Italy (Table 5). These refer to test or comparator substances, rather than unique trials (n = 1,051), and may be counted multiple times within a single study due to variations in dosage or formulation. Chemical IMPs dominated the dataset (n = 2,497; 66.1%), and experienced a notable rise between 2019 and 2022, peaking at n = 390 in 2021. Biological IMPs followed (n = 1,082; 28.6%), steadily increasing until 2020 and then declining slightly in 2023. ATMPs were fewer (n = 127; 3,4%) peaking in 2020 and 2021 (n = 19 and n = 21, respectively). Mixed classifications, such as chemical/biological (e.g., antibody-drug conjugates) (n = 71; 1.9%) remained marginal showing a consistent trend throughout the years.

**TABLE 5:** Annual Distribution of IMPs by Category. This table presents the yearly distribution of IMPs included in Phase I clinical trials submitted in Italy between 2015 and January 2025 (N = 3,777), categorized as Chemical, Biological, Advanced Therapy Medicinal Products (ATMPs), or combinations of Chemical and Biological agents. For each year, absolute numbers (n) and percentages (%) are reported. (*)The Total IMP percentages refer to the cumulative distribution over the entire observation period.

Year	Chemical		Biological		ATMP		Chemical, biological		Total	
	n	%	n	%	n	%	n	%	N	%
2015	184	72.4	60	23.6	5	2.0	5	2.0	254	100
2016	208	66.0	98	31.1	6	1.9	3	1.0	315	100
2017	196	61.6	103	32.4	12	3.8	7	2.2	318	100
2018	149	49.3	129	42.7	17	5.6	7	2.3	302	100
2019	256	69.2	102	27.6	10	2.7	2	0.5	370	100
2020	296	61.8	156	32.6	19	4.0	8	1.7	479	100
2021	390	69.6	139	24.8	21	3.8	10	1.8	560	100
2022	360	73.2	111	22.6	10	2.0	11	2.2	492	100
2023	209	67.9	78	25.3	15	4.9	6	1.9	308	100
2024	244	66.7	101	27.6	12	3.3	9	2.5	366	100
Jan 2025	5	38.5	5	38.5	0	0.0	3	23.1	13	100
Total IMP (2015-January 2025) ^(*)^	**2,497**	**66,1**	**1,082**	**28,6**	**127**	**3,4**	**71**	**1,9**	**3,777**	**100**

#### 3.7.2 Distribution by phase I study classification

**FIGURE 8:**
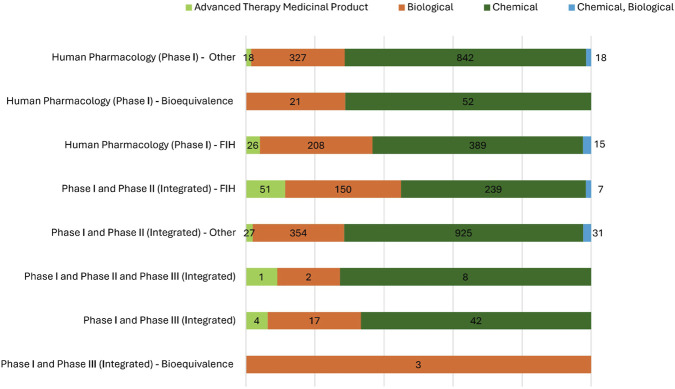
Distribution of IMP categories across Phase I studies in Italy. This figure illustrates the distribution of different IMP categories—Chemical, Biological, Advanced Therapy Medicinal Products (ATMPs) and hybrid (Chemical, Biological)—are distributed among various Phase I trials, including standard exploratory studies, bioequivalence studies, First-In-Human (FIH) studies, and integrated Phase I/II or I/III models.

Different product categories were associated with distinct Phase I trial classifications and designs (Figure 8).

Chemical IMPs (n = 2,497/3,777; 66.1%) were particularly prevalent in integrated Phase I/II–Other trials (n = 925; 37.0% of all chemical IMP trials) and Human Pharmacology–Other trials (n = 842; 33.7% of all chemical IMP trials).

Biological IMPs (n = 1,082/3,777; 28.6%) were tested especially in integrated Phase I/II–Other studies (n = 354; 32.7% of all biological IMP trials), in Human Pharmacology–Other (n = 327; 30.2% of all biological IMP trials) and in FIH trials (n = 208; 19.2% of all biological IMP trials). The small number of bioequivalence trials involving biologics (n = 24; 2.2%) is consistent with their limited use in comparative pharmacokinetics.

ATMPs, while less numerous (n = 127/3,777; 3.4%), showed a clear preference for integrated FIH studies (n = 51; 40.2% of all investigational ATMP trials).

Hybrid chemical-biological IMPs, though infrequent (n = 71/3,777; 1.9%), were most often studied in integrated Phase I/II (n = 31; 43.7%) and FIH (n = 22; 31.0%) designs.

#### 3.7.3 Distribution across participant populations

IMP use differed by population type. Chemical products (n = 2,497/3,777) were predominantly tested in trials involving both patients and vulnerable groups (n = 2,148; 86.0%) but also appeared in studies with healthy volunteers (n = 46; 1.8%).

Biologics (n = 1,082/3,777) had broader population targeting, with n = 30 (2.8%) entries for healthy-only subjects and n = 17 (1.6%) mixed volunteer and patient cohorts, though the majority (n = 43; 87.2%) were tested in patient with vulnerable groups. ATMPs (n = 127; 3.4%) were almost exclusively tested in patient and vulnerable populations (n = 113; 88.9%). Only n = 14 (11%) was used in non vulnerable patient studies. Chemical/biological IMP were rarely tested in HVs, with just one entry (n = 1/71; 1.4%).

#### 3.7.4 Distribution by sponsor typology

A significant imbalance in sponsor distribution emerged in the IMP categories. Most Chemical IMPs (n = 2,319; 92.8%) were submitted by commercial sponsors, while non-commercials contributed n = 178 (7.2%). The number of commercial chemical IMPs peaked in 2020 and 2021 (n = 357 and n = 343, respectively). A similar pattern was observed for Biological IMPs: n = 1,040 entries (96.1%) were submitted by commercial sponsors, with just n = 42 entries (3.9%) submitted by non-commercial sponsors across the decade. Commercial activity peaked in 2020 and 2021 (n = 138 and n = 135 entries, respectively), then dipped slightly.

ATMPs displayed a more balanced distribution of sponsors: n = 85 (66.9%) commercial vs. n = 42 (33.1%) non-commercial entries. Hybrid categories were rare but insightful: n = 3 out of the 71 chemical/biological entries were submitted by non-commercial sponsors, demonstrating their engagement in innovative research despite structural constraints.

## 4 Discussion

This ten-year analysis of Phase I clinical trials submitted in Italy provides an in-depth view of the evolution of early clinical development within a changing regulatory and competitive landscape.

Although Western Europe retained the largest overall share, its contribution to global trial-country utilization fell by 12% between 2019 and 2024. Meanwhile Central and Eastern Europe’s share declined by 28% (IQVIA Institute for Human Data Science, 2025), despite an increase in global clinical research volumes increased of 38% (IQVIA-EFPIA-VE, 2024). Phase I trials were particularly affected, with sponsors increasingly favouring regions offering faster start-up timelines and more predictable regulatory pathways. Notably, pre-commercial biotech companies initiated more than twice as many Phase I trials as large pharmaceutical companies in 2024 (IQVIA Institute for Human Data Science, 2025), confirming a broader shift in innovation towards agile, technology-driven organizations.

While Italy has shown a degree of resilience, its overall contribution to Phase I research remains limited when compared to other leading European countries, as evidenced by comparative data (Di Tonno et al., 2022). Despite the gradual increase in self-certified Phase I units under AIFA Determination 809/2015 during the 2015–2025 decade (AIFA, 2025b), A persistent difficulty in fostering early-phase development remains evident. Without targeted initiatives to foster innovation and attract high-value trials, the country risks being marginalized in this strategically important segment.

Despite these concerns, Italy maintained a relatively stable volume of Phase I activity over the ten-year period from January 2015 to the end of January 2025, encompassing the full transition from the EU Clinical Trials Directive to the Clinical Trials Regulation (EU) 536/2014, with notable peaks in 2021 and 2022. These peaks may reflect the dual impact of the 2020–2022 SARS-CoV-2 pandemic (Audisio et al., 2022; Cavaleri et al., 2021) and the transitional pressure of Regulation prior to the rollout of *Accelerating Clinical Trials in the European Union* (ACT EU) (Capone et al., 2024; De Mey, 2022), the joint initiative launched by the European Commission, EMA, and HMA in 2022 to strengthen the attractiveness of Europe for clinical research by improving regulatory efficiency, enhancing transparency, and fostering patient and stakeholder (HMA-EC-EMA, 2022).

While the absolute number of Phase I trials has remained relatively stable, their proportion relative to the total number of clinical trials increased over time. This divergence highlights a methodological consideration: while the absolute number of Phase I trials remained reasonably steady, their increasing proportion among all clinical trials may reflect a relative decline in later-phase submissions, rather than a genuine expansion of early development capacity. Although this hypothesis was not formally tested, it stems from the observed trends in the registry data and aims to contextualize the findings within the evolving national research landscape.

The progressive implementation of quality-focused regulatory frameworks, most notably AIFA’s Determina 809/2015, has resulted in more stringent requirements being set for Phase I units. These requirements emphasize risk management, emergency preparedness, and integrated quality systems (Di Tonno et al., 2024). These measures should contribute to improving the scientific and operational standing of Italian centres and support renewed engagement in early development (Cagnazzo et al., 2020). However, regulatory advancement alone has not led to a proportional increase in trial activity, likely due to structural limitations, such as limited public funding, bureaucratic complexity, and insufficient investment in academic research infrastructure. The data presented in this analysis reveal several notable characteristics of the Italian Phase I research landscape. The sponsor typology further clarifies the structural characteristics of early development in Italy. Industry dominates across all therapeutic areas, with non-commercial sponsors accounting for less than 10% of total trials. These sponsors often face resource constraints, regulatory hurdles, and operational challenges when adapting to platforms such as CTIS. Moreover, previous observations (Dri et al., 2025) suggest that studies sponsored by non-commercial entities are more likely to be withdrawn or rejected, highlighting the necessity of targeted support mechanisms. Academic engagement is lacking for some therapeutic areas, such as Skin and Connective Tissue diseases and particularly limited in Oncology, Congenital, Hereditary, Neonatal diseases and Cardiovascular diseases, which are almost exclusively explored by commercial entities. A few domains—such as Nutritional/Metabolic diseases, Immune System diseases, and Hematologic diseases—show relatively higher non-commercial participation. However, the complete absence of non-commercial Phase I activity in nine therapeutic categories highlights the fragility of academic-led early development.

Population strategies also reveal important patterns. A significant proportion of the analyzed Phase I trials included participants who were classified as vulnerable. More than 80% of the trials included information regarding specific vulnerability categories, confirming that their inclusion is a consistent feature of early development in Italy. This challenges the assumption that vulnerable groups are systematically excluded from early-phase trials (Binik, 2024; Shepherd, 2020; Shepherd et al., 2019; Waltz et al., 2023). Conversely, these groups are frequently involved, particularly in studies led by non-commercial sponsors. Notably, trials sponsored by academic or institutional entities were more likely to include participants incapable of and enroll subjects falling into multiple vulnerability categories. These findings reinforce the critical role of academic and institutional sponsors in promoting equitable access to research for underrepresented or excluded populations (Bourgeois et al., 2012; Shepherd, 2020), often without the financial incentives driving industry-sponsored studies. Although the 2024 revision of the Declaration of Helsinki (WMA, 2024) was released after the observation period of this study, the trends observed in the Italian dataset appear to anticipate several of its key ethical priorities. The updated Declaration places renewed emphasis on the inclusion and protection of vulnerable groups, particularly in early-phase research where risks and uncertainties are higher. This suggests that, despite structural constraints, Italy has contributed to shaping a more inclusive and ethically aware research landscape in the early stages of development.

Further analysis of participant characteristics revealed a marked underrepresentation of healthy volunteers (HVs), despite their foundational role in early clinical pharmacology (Pasqualetti et al., 2010; Sramek et al., 2021). In our dataset, only 3.4% of Phase I trials enrolled HVs exclusively. Comparative data from ClinicalTrials.gov underscore this gap: between January 2015 and January 2025, just 10 HV-only Phase I studies were registered in Italy, versus 317 in Germany and over 200 in both the Netherlands and Belgium (personal dataset extracted from ClinicalTrials.gov on 5 June 2025). Multiple factors may explain this discrepancy, including the absence of a national HV registry, limited public awareness, and operational inefficiencies in recruitment and trial design (Allen et al., 2017; Boyce et al., 2013; Resnik, 2011). We hypothesize that economic drivers—such as cost-efficiency and faster execution timelines—substantially shape the geographic distribution of HV Phase I trials, particularly for studies with low methodological complexity. The predominance of commercial sponsors in our dataset supports this notion, suggesting that early-phase trials not requiring highly specialized infrastructure are often placed in countries with more competitive HV trial ecosystems. Furthermore, the EU’s requirement for quality dossier for each non-authorized IMP may discourage early-phase research within its borders, as sponsors often prefer jurisdictions with less stringent entry requirements for first-in-human or exploratory studies. Bridging these gaps in Italy will require healthcare policies aimed at ensuring predictable costs and timelines for clinical trial approval and conduct, strategic investment in structured HV databases, public outreach campaigns, and targeted support for HV trials led by academic and non-commercial stakeholders.

Another potential factor contributing to the limited presence of Phase I trials in Italy may relate to structural changes within the CRO landscape. Over the past decade, the European CRO sector—Italy included—has undergone significant consolidation. Many smaller or regionally based providers have merged into large multinational organizations offering integrated services that span from early-phase design to regulatory submission and site management. This evolution, widely documented in industry analyses and strategic reports (ProClinical, 2025; Clinical Trials Arena, 2022), has reduced the number of independent early-phase CROs operating in Europe. Although this phenomenon is not directly observable within the regulatory registries analysed in this study, we hypothesize that it may indirectly influence the localization and operational feasibility of Phase I trials. Larger CROs, with their enhanced capacity to manage complex regulatory environments and to implement technological innovations—such as decentralized clinical trials or centralized feasibility platforms—may steer sponsors toward trial locations offering more scalable and cost-efficient infrastructures. As a result, countries with higher CRO integration, digital capability, and optimized regulatory processes may become more attractive for the conduct of early development studies.

In terms of therapeutics, the Italian Phase I landscape remains heavily concentrated in oncology, which accounts for nearly 70% of all submitted trials. While this reflects global pharmaceutical development priorities, it also highlights a lack of diversity, with other high-burden disease areas such as cardiovascular, respiratory, and metabolic diseases receiving limited attention. This imbalance raises questions about Italy’s capacity to support diversified early-phase research that is aligned with broader public health needs.

Age stratification shows a clear predominance of adult and elderly populations, with limited inclusion of children and adolescents. Even within oncology, only 8.6% of trials included pediatric subjects. This trend persists despite the requirements introduced by Regulation (EC) No 1901/2006 (EU, 2006) and the Paediatric Investigation Plan (PIP) framework, suggesting ongoing challenges in pediatric trial design and feasibility. A systematic review of early-phase pediatric clinical pharmacology trials further confirms that infants, children, and adolescents are often excluded or minimally represented in Phase I studies—highlighting the ethical and logistical barriers that continue to hinder pediatric participation in early development (Subramanian et al., 2022).

Phase I trials demonstrate broad inclusion of both sexes, with the vast majority of studies enrolling both. However, sex-specific analyses remain rare. This aligns with persistent evidence of underrepresentation and limited stratification by sex in early-phase trials (Douthard et al., 2022; Liu and DiPietro Mager, 2016; Zucker and Prendergast, 2020). These highlight the importance of systematically integrating sex-based variables into the early stages of human pharmacology.

Analysis of IMPs confirms a traditional predominance of chemical entities but also reflects the growing role of biologics and the structured emergence of ATMPs. Biologics have become increasingly prevalent in integrated and FIH trials, while ATMPs were most frequently tested in integrated designs and vulnerable populations.

Between 2015 and 2021, the number of ATMP trials increased by over 300%, particularly in non-commercially sponsored studies focusing on across rare diseases and neurology. This trend, however, declined after 2021, raising concerns about its sustainability in the absence of adequate infrastructure and strategic engagement with EU-level acceleration tools such as the PRIME scheme (EMA, 2025b) and the ACT EU Workplan 2025–2026 (HMA-EC-EMA, 2024). EMA’s publication of the 2025 guideline on investigational ATMPs (EMA-CAT, 2025) recognizes the complexity of these products, calling for tailored approaches to trial design and regulatory interaction. Although still limited in absolute numbers, Phase I trials involving ATMPs appear to show a gradual upward trend. This reflects a broader evolution of early-phase research (EMA-CAT, 2025): Italian ATMP trials reflect many of these principles, but their limited transition to later-phase development highlights the need for stronger national translational pathways.

Taken together, these findings depict a Phase I research ecosystem that is scientifically mature yet structurally constrained. To achieve a more balanced and resilient model, dedicated support is needed for non-commercial research, investments in infrastructures are required, and greater integration with EU strategies such as ACT EU and Regulation (EU) 536/2014 is necessary. Without such alignment, Italy risks losing competitiveness in the early phases of drug development.

## 5 Strengths and limitations

This study offers a structured and comprehensive overview of Phase I clinical trial submissions in Italy over a ten-year period, integrating data from national and European regulatory platforms (OsSC, EudraCT, and CTIS). One of its main strengths lies in the use of official and validated datasets, aligned with AIFA’s classification systems, which enhances the robustness and comparability of the analyses. The inclusion of the entire regulatory transition period—from Directive 2001/20/EC to Regulation (EU) No. 536/2014—adds further relevance and timeliness.

Some limitations, however, must be acknowledged. The data were retrieved from three distinct platforms that differ in architecture and data-entry fields, requiring retrospective harmonization. Moreover, all information was provided by sponsors and may vary in accuracy or completeness. Specific variables—such as the role of applicants or detailed IMP classification—were not always consistently reported, particularly under the new Regulation. Importantly, the analysis reflects regulatory submissions and does not capture actual trial conduct or outcomes. In some instances, the retrospective classification of study design required interpretative decisions.

Despite these constraints, this study represents the first comprehensive attempt to map Phase I trial activity in a European Member State across both legislative frameworks. It offers a valuable foundation for monitoring future trends and informing both national policy and European-level planning.

## 6 Conclusion

This overview of Phase I clinical trials in Italy outlines a stable system that has gradually integrated scientific and regulatory innovations. The increasing importance of early-phase studies indicates a change in the national research portfolio, although this does not necessarily imply an increase in capacity.

Key findings highlight areas of progress and limitation. The dominance of oncology and commercial sponsorship reflects consolidated priorities, while the limited presence studies involving healthy volunteers and non-commercial activity in several therapeutic areas indicates untapped potential. The increasing role of biologics and ATMPs signals evolving competencies, though challenges remain in sustaining and translating these efforts.

Notably, vulnerable participants were frequently included, particularly in academic trials. Although there was a good balance or high inclusion of both sexes, sex-specific analyses were uncommon. These patterns highlight the importance of methodological refinement and inclusive research strategies.

Future efforts should focus on strengthening infrastructure for non-commercial research, improving frameworks for healthy volunteer engagement, and supporting continuity in advanced therapy development. Further monitoring will be essential to understand how recent policy and regulatory changes may influence Italy’s early-phase research trajectory. In parallel, a dedicated comparative study across EU Member States is warranted to benchmark national capacities, identify best practices, and assess the impact of ACT EU and other harmonization initiatives on the competitiveness of early-phase research across Europe.

## Data Availability

Aggregated data used in this study were obtained through structured queries under the supervision of AIFA and are not publicly available in their entirety due to regulatory confidentiality constraints. Requests for access to the dataset should be addressed directly to AIFA.

## Footnotes

1 Medical Subject Headings (MeSH). U.S. National Library of Medicine. Available at: https://www.ncbi.nlm.nih.gov/mesh

2 The classification of Investigational Medicinal Products (IMPs) by type (e.g., chemical, biological, ATMP) was performed using tailored approaches to account for structural differences between the two registries. As the OSSC platform and the CTIS portal employ different data architectures-particularly with respect to the fields specifying IMP categories- IMPs were classified retrospectively based on the product name and characteristics, in accordance with the classification criteria adopted by the OsSC system. This harmonized methodology ensured consistency and comparability across the complete dataset covering the period 2015–2025 dataset despite variations in data structure between the sources
